# The need for sustainable innovation in an era of political uncertainty

**DOI:** 10.1093/eurpub/ckaf021

**Published:** 2025-04-03

**Authors:** Tit Albreht, Charlotte Marchandise, Ilmo Keskimäki, Floris Barnhoorn

**Affiliations:** President of EUPHA; Executive Director of EUPHA; Chair of the 18th EPH Conference; Deputy-Director EPH Conference

The global public health community is facing a new era of uncertainty. As the USA embarks on another political shift, the risks to global health initiatives, scientific collaboration, and evidence-based policymaking are mounting. The lessons of the past teach us that public health can be an easy target in times of political upheaval, particularly when misinformation and disinformation infiltrate public discourse. As Professor Martin McKee from EUPHA warns, “disinformation is the new normal with far-reaching implications for society, including population health.” The scientific community must be proactive, not reactive, in confronting these challenges. Our tools—science, advocacy, and capacity building—are necessary but insufficient. Public health must also embrace innovation across multiple domains while remaining connected to our core principles of equity and sustainability.

Public health has long been the invisible force safeguarding societies from disease, environmental hazards, and structural inequities. However, in an era of algorithm-driven news cycles and entertainment-dominated attention spans, public health must evolve beyond traditional modes of communication. We must foster new pathways of innovation to ensure the resilience and adaptability of our systems in the face of rapid global change.

Innovation in public health must take multiple forms—digital, social, low-tech, green, and cultural. Digital innovation, including AI-powered analytics and mobile health applications, can help improve accessibility and real-time monitoring. Social innovation, through participatory health models and behavior change strategies, strengthens community resilience. Low-tech solutions ensure that underserved populations are not left behind, offering cost-effective and scalable approaches. Green health solutions, such as nature-based interventions and sustainable healthcare systems, support planetary health. Finally, cultural innovation is a crucial but often overlooked pathway—leveraging media, art, and storytelling to shape public understanding, combat misinformation, and build trust in public health efforts. EUPHA will continue supporting innovative and comprehensive communication in response to the mentioned challenges.

EUPHA is committed to fostering a stronger public health voice through initiatives like the European Public Health Week (EUPHW), which will take place from 13–17 May 2025. This annual event serves as a platform to engage diverse audiences, strengthen collaborations, and ensure that public health remains a visible and urgent priority. The theme for EUPHW 2025 will focus on innovation, resilience, and equity, reflecting the need to address public health challenges in a rapidly changing world.

EUPHW also presents an opportunity to bring to life the Lisbon Statement from the 2024 Lisbon EPH Conference at the national and local levels. By engaging partners and stakeholders across Europe, we can ensure that the statement is not merely a symbolic declaration but is translated into meaningful action and impact. EUPHW provides the momentum to align advocacy efforts, foster concrete commitments, and integrate the conference’s key messages into policies and practices across different countries, creating a movement for the years to come.

We want to highlight how public health professionals, policymakers, and communities can collectively shape resilient and sustainable health systems. This moment presents an opportunity to engage a wider audience, create meaningful dialogues, and advance innovative solutions that address pressing global health challenges. It offers a straightforward, dynamic, and inclusive platform where anyone can contribute ideas to make science more accessible—whether in English or their native language, through in-person discussions or online. In that sense, EUPHA remains inclusive and open to collaboration to all public health professionals and researchers in the WHO European region, but also beyond in global collaborations for improved planetary health.

As we navigate these turbulent times, one truth remains: public health is political, cultural, innovative, and deeply human. We must step beyond our conventional boundaries, make our voices heard in society, and reaffirm our commitment to protecting health as a fundamental right for all. The stakes have never been higher, and our response must be bold, creative, and unwavering.

To learn more and engage in the European Public Health Week https://eupha.org/euphw

## Welcome to the 18th European Public Health Conference

KeskimäkiIlmo
Chair of the 18th EPH Conference
BarnhoornFloris
Deputy-Director EPH Conference

The 18th European Public Health (EPH) Conference will take place in “Helsinki, Finland, from 11–14 November 2025.” Pre-conferences are scheduled for Tuesday, 11 November, followed by the main conference from 12 until 14 November. The theme for 2025 is: “Investing for Sustainable Health and Well-Being.”

### Investing for sustainable health and well-being

In recent years, Europe has faced significant challenges, with many governments losing sight of crucial issues for their countries and the world. In too many parts of the world, war and violence have re-emerged, causing immense suffering and becoming major public health threats. While peace efforts are self-evidently the most urgent public health tasks, other critical public health issues have also been neglected far too long. Overwhelming evidence shows that ecological and social sustainability is essential for the future of our planet and humanity. However, despite knowing what needs to be done to achieve this, changing outdated economic, social, and health policies remains difficult. The upcoming EPH Conference emphasizes this need for societal change.

As expressed in our conference theme “Investing for sustainable health and well-being”, health should be seen as an investment, not an expense. By promoting integrated and sustainable health approaches, the conference aims to inspire policies and practices that support health equity, environmental sustainability, and social justice. Investing in health and well-being is crucial for building resilient communities and a sustainable future. This article addresses one of the sub-themes of the conference “Economy of well-being and public health,” highlighting their interconnectedness and the benefits of investing in sustainable health and well-being.

### Economy of well-being and public health

Investing in population health and well-being is a strategic investment that yields significant societal returns. It fosters a sustainable economy by enhancing productivity, reducing healthcare costs, and promoting social stability. Health and well-being are crucial for sustainable development, supporting social, economic, and environmental sustainability. A healthy planet with inclusive growth and fair societies enhances individual and community well-being. Addressing health determinants and reducing inequities are essential for achieving population health and sustainable development. Health investments bring numerous societal and economic benefits. Healthier populations are more productive, leading to increased economic output. Preventive healthcare reduces chronic diseases and long-term healthcare costs. Improved health outcomes contribute to social cohesion and stability, making communities more resilient to economic and environmental changes. Investing in mental health promotion enhances quality of life and educational outcomes. Health investments also support progress toward achieving multiple Sustainable Development Goals, including poverty reduction, education, gender equality, and climate action. Access to quality healthcare reduces poverty by preventing health-related financial hardships. Healthier children perform better academically, and gender equality is promoted through health services addressing women’s and girls’ needs. Health investments focusing on environmental sustainability, such as reducing pollution and promoting clean energy, contribute to a healthier planet and mitigate climate change impacts.

The economy of well-being emphasizes the interconnectedness of the economy, social security, healthcare, planetary boundaries, and overall welfare, focusing on long-term sustainability. It prioritizes people’s well-being in policymaking, recognizing that economic growth alone does not equate to improved quality of life. Instead, it advocates for policies that enhance health, social equity, and environmental sustainability, ensuring that economic activities contribute positively to societal welfare. It is evident that current economic systems, which prioritize GDP growth, often contribute to environmental crises, social inequalities, and rising noncommunicable diseases. These systems fail to generate true well-being as they overlook the negative externalities of economic activities, such as pollution, resource depletion, and health disparities. Addressing these issues requires a paradigm shift from a growth-centric model to one that measures and promotes well-being through integrated and consistent policies. This can be achieved by moving beyond the GDP (Gross domestic product) to include alternative metrics, which better capture the multifaceted nature of societal progress. Implementing a Health in All Policies (HiAP) approach ensures that health considerations are integrated into all sectors, from transportation to education, promoting policies that support health and well-being. By shifting to an economy of well-being, societies can achieve sustainable development that prioritizes health, equity, and environmental stewardship, ultimately leading to a higher quality of life for all.

### Call for abstracts

Abstract submission for Helsinki 2025 is open until 1 May 2025 (18:00 CET). Submit your work for workshops, oral presentations, pitch presentations, or ePosters. Don’t miss this opportunity to showcase your hard work, learn from others, and share your knowledge. More information here

### Helsinki, Finland

Helsinki, the capital of Finland, combines urban culture, serene nature, a stunning archipelago, and vibrant urban areas. The city’s compact size makes navigation easy, allowing extensive exploration during your stay. Helsinki is known as a safe, secure, and clean city where international congresses are run with enthusiasm, with an emphasis on sustainability, attention to the human dimension, and a generous degree of hospitality.

We look forward to welcoming you to Helsinki!

